# Type 2 diabetes in the employed population: do rates and trends differ among nine occupational sectors? An analysis using German health insurance claims data

**DOI:** 10.1186/s12889-024-18705-5

**Published:** 2024-05-03

**Authors:** Batoul Safieddine, Julia Grasshoff, Siegfried Geyer, Stefanie Sperlich, Jelena Epping, Johannes Beller

**Affiliations:** Medical Sociology Unit, Hannover Medical School, Hannover, Germany

**Keywords:** Type 2 diabetes, Occupational sector, Employment, Trends, Health insurance claims data, Germany

## Abstract

**Background:**

Socioeconomic inequalities in type 2 diabetes (T2D) are well established in the literature. However, within the background of changing work contexts associated with digitalization and its effect on lifestyle and sedentary behavior, little is known on T2D prevalence and trends among different occupational groups. This study aims to examine occupational sector differences in T2D prevalence and trends thereof between 2012 and 2019.

**Methods:**

The study was done on 1.683.644 employed individuals using data from the German statutory health insurance provider in Lower Saxony, the “Allgemeine Ortskrankenkasse Niedersachsen” (AOKN). Predicted probabilities for T2D prevalence in four two-year periods between 2012 and 2019 were estimated based on logistic regression analyses for nine occupational sectors. Prevalence ratios were calculated to illustrate the effect of time period on the prevalence of T2D among the nine occupational sectors. Analyses were stratified by gender and two age groups.

**Results:**

Results showed differences among occupational sectors in the predicted probabilities for T2D. The occupational sectors “Transport, logistics, protection and security” and “Health sector, social work, teaching & education” had the highest predicted probabilities, while those working in the sector “Agriculture” had by far the lowest predicted probabilities for T2D. Over all, there appeared to be a rising trend in T2D prevalence among younger employed individuals, with gender differences among occupational sectors.

**Conclusion:**

The study displayed different vulnerability levels among occupational sectors with respect to T2D prevalence overall and for its rising trend among the younger age group. Specific occupations within the vulnerable sectors need to be focused upon in further research to define specific target groups to which T2D prevention interventions should be tailored.

**Supplementary Information:**

The online version contains supplementary material available at 10.1186/s12889-024-18705-5.

## Background

Type 2 diabetes (T2D) is a widely prevalent and growing chronic disease identified as a rising global epidemic [1]. In Germany, overall prevalence rates are reported to range between 7 and 12% [2, 3], and future projections estimate an increase of 54–77% in the number of individuals with T2D between the years 2015 and 2040 [4]. This has significant ramifications on the public health burden as reverberated by expansion of morbidity [5], effects on healthy aging [6], deteriorated occupational health [7] and rising economic costs [8].

Among the major risk factors of T2D are lifestyle habits associated with overweight and obesity, an outcome that has been on the rise during the last few decades [9]. Nutrition and physical activity are key factors when it comes to risk factor management of T2D. In fact, planning risk factor management interventions requires targeting individuals in several contexts of their daily lives. Especially in a country like Germany where around 77% of individuals aged 15–65 years belong to the working population [10], the occupation and work environments are important contexts for T2D risk factor management and prevention. In a recent analysis on trends of sitting time in Europe, results indicated a significant increase in sitting time in individuals aged 21–65 years, which is the age range for most working individuals [11]. Moreover, obesity trends have been on the rise in Germany especially among middle-aged individuals [12]. Temporal changes in occupational contexts such as digitalization, which might be associated with an increased use of technology instead of manual work as well as less commute due to the increased availability of digital communication technologies and possibilities for working from home [13], might very well carry alongside increased risks for non-communicable diseases. Therefore, one important population subgroup to focus on when examining the highly lifestyle-dependent T2D prevalence and trend is the employed population, for which specific vulnerable subgroups should be identified.

Socioeconomic inequalities in T2D have been well established in the literature. Evidence from industrialized countries points towards inequalities where individuals of lower SES, as depicted by education, income and occupation, are more affected [14–17]. Most commonly however, studies consider occupational class that reflects occupational prestige, autonomy and qualification to depict occupational inequalities [18]. Nevertheless, even though very scarce, evidence points towards inequalities in the risk of T2D among different occupational groups regardless of social class [19–21]. Moreover, evidence shows that the risk of mortality is higher in individuals with T2D that work in certain occupational groups compared to others [22]. In fact, T2D can be associated with workplace risk factors such as prolonged sitting time, which would be the case for several occupations that vary greatly in class and autonomy. Moreover, several occupational exposures that might differ widely among occupations, regardless of occupational class, can be associated with a metabolic risk profile in working individuals [23, 24]. For example, nutritional factors associated with different occupational contexts in terms of availability of healthy food choices at work or challenges related to working times play a major role in building the risk profile of individuals [25, 26]. Among other occupational exposures is shiftwork, which is a working parameter common among health care providers, security personnel or even factory workers, posing them to challenges associated with sleep routines, stress, and eating habits [27, 28]. Besides that, some occupations expose workers to certain chemicals that induce oxidative stress, posing individuals to higher metabolic risks [29]. In addition, stress is an important factor within this context, as high-stress occupations have been shown to have prominent stress-induced metabolic risk profiles [30, 31].

Despite rising trends in metabolic risk factors and differences in occupational exposures among different occupational contexts, only a few studies considered occupational groups or sectors imposing a research gap within this field and to our knowledge, no evidence on occupational sector differences in T2D exists for Germany. Determining vulnerable occupational fields would help target T2D prevention interventions where they should be at, in order to improve occupational as well as overall health. This study aims to participate in bridging this gap by examining the prevalence and the trends of T2D among nine broad occupational sectors. This would help identify vulnerable occupational sectors that need to be focused upon to identify specific occupations to which T2D prevention interventions should be tailored. Moreover, against the background of the rising trends of occupational digitalization and prolonged sitting time [11, 13], understanding the trends of T2D prevalence in different occupational sectors would better inform health care policy aiming at improving occupational health. Precisely, using claims data of a large German statutory health insurance provider, this study aims to identify vulnerable occupational groups by:

1. examining the rates of T2D among different occupational sectors of employed individuals.
2. examining the trends of prevalent T2D in different occupational sectors.

## Methods

### Database

The study was performed using data from the largest statutory health insurance provider in the state of Lower Saxony, Germany: the “Allgemeine Ortskrankenkasse Niedersachsen” (AOKN). While health insurance is mandatory for all residents in Germany, AOKN insures around one third of the population in Lower Saxony, forming a population size of approximately 3 million men and women [32]. In the German statutory health insurance system, insurance premiums depend on individual income, and medical services are offered equally to all insured individuals. The data includes all in- and outpatient diagnoses coded according to the German-modified tenth version of the international classification of diseases (ICD-10-GM). In addition, all medical treatments and prescribed medications are registered. The data also includes socioeconomic information on education, individual income and occupation as provided by the employers who are legally bound to submit certain information within the social security system. The data is available for the years 2005 to 2019.

### Ethical approval

This study did not require ethical approval, as it involved a pre-existing claims dataset created as part of the routine administrative activities of AOKN. Its scientific use is regulated by German law in the German Social Code “Sozialgesetzbuch”. The data protection officer of the Local Statutory Health Insurance of AOKN has given permission for this study to use the data for scientific purposes.

### Occupational sector

Occupation was operationalized according to the corresponding occupational sector as classified by the most current classification of occupations (KldB2010), issued by the German Federal Employment Agency in 2010 [33]. KldB2010 entails a more detailed classification and better matches the international standard classification of occupations (ISCO) compared to the previous versions. Occupational codes from the previous versions (about 330 codes) cannot be matched well to the current classification of occupations (about 1280 codes). Consequently, the study was confined to employed individuals insured in the years between 2012 and 2019, for which occupation is coded according to the new classification KldB2010. KldB2010 entails 5-digit codes for specific occupations, representing more than 1280 occupations belonging to 10 major occupational sectors. These ten occupational sectors are: “Military”, “Agriculture”, “Extraction of raw material, production and manufacturing”, “Construction, architecture, measuring and building technology”, “Natural sciences, geography, information”, “Transport, logistics, protection and security”, “Commercial, trade, distribution and tourism”, “Corporate organization, accounting, law and administration”, “Health sector, social work, teaching & education” and “Humanities, culture and design”. The occupational sector “Military” was excluded from the analyses due to the very small sample size, since health care costs of individuals working in this sector are usually covered by federal aid (Beihilfe) and private insurance. The study examined rates and trends of T2D among the nine remaining occupational sectors. For individuals which had several occupational sectors within each period, the occupational sector of the longest duration was considered.

### Age groups

The analyses in this study were stratified by two age groups: 18–45 years and 46 years or older. This choice was based on several considerations. First, the age of 45 years is considered the threshold for the onset of T2D [34], as early-middle age is associated with physiological changes that increase T2D risk [35, 36]. Second, different stages of adulthood are associated with distinct social and biological challenges that influence risk profiles and intervention approaches [37]. The influence of occupation on health may also vary depending on these life stage-associated challenges. Although analyzing early adulthood (18–35 years) separately would have been ideal, it would have led to insufficient case numbers in some strata due to the multi-level stratification applied. Therefore, a compromise was to split the age groups at 45 years, which is interesting as it coincides with the stage of raising children, as the average age of having a first child in Germany ranges between 30–33 years [38, 39]. Thus, this split allows for examining the unique social and familial challenges that working individuals face in each life stage and how these challenges may influence their lifestyle, well-being, and health differently.

### Definition of T2D

The definition of T2D was done similar to previous publications using the same database [5, 14, 40, 41]. In each time period, individuals who were insured for more than one quarter were considered to have prevalent T2D if they had valid T2D diagnoses in at least two quarters. We did not apply this condition to individuals who were insured for only one quarter in the specified time period, i.e. individuals insured for only one quarter and who have valid T2D diagnoses in that quarter were also defined to have prevalent T2D.

The ICD-10-GM codes for Diabetes mellitus range between “E10” and “E14”, with different numbers between “10” and “14” corresponding to the type of Diabetes mellitus. The code “E11” refers to T2D. However, the data are associated with some coding errors, as several diabetes codes (codes corresponding to different types of diabetes) were sometimes coded for the same individual in the same time period. Therefore, in our study, T2D diagnoses were considered to be valid based on some considerations. In each time period, individuals were considered to have a valid T2D diagnosis if:

1. Among other coded “E” codes, “E11” was coded most frequently in the specified time period. Or,
2. “E14”, which refers to “undefined type of diabetes”, was most frequently coded in the specified time period (since T2D represents the vast majority of diabetes cases (around 90%)). Or,
3. “E10”, which refers to type 1 diabetes, was coded most frequently, but insulin was not prescribed in the same specified time period (since type 1 diabetes also requires insulin prescriptions).

### Time periods

The rates and trends of T2D were examined over four two-year time periods between 2012 and 2019 as follows: 2012–2013 (p1), 2014–2015 (p2), 2016–2017 (p3) and 2018–2019 (p4). In order to limit sources of bias and make the time periods more comparable by sharing similar sources of potential errors, occupational sector as well as T2D were defined in each time period anew according to the above-mentioned criteria.

### Statistical analyses

The two-year prevalence rates of T2D (in each time period) in employed individuals and among the different occupational sectors were illustrated by predicted probabilities which were based on logistic regression analyses. Predicted probabilities are estimates of the prevalence that take into account adjusted covariates [42]. For all employed individuals, models were stratified by the two mentioned age groups and gender (four strata). As a next step, Models were stratified by age group, gender and the nine occupational sectors, resulting in 36 strata. In all models, the dichotomous variable “prevalent T2D” (0 “no”, 1 “yes”) was the dependent variable. “Time period” with the four categories p1, p2, p3 and p4 was the main independent variable, with p1 as the reference group. All models adjusted for the covariates “Age” and “Number of days insured”: “Age” in years was added as a continuous variable and refers to the age within each strata (age in years within the corresponding age group), as we have two age groups with seemingly wide ranges. “Number of days insured” was a continuous variable and corresponds to the number of days individuals were insured within each two-year time period. It was added to the models in order to adjust for potential censoring, because the longer individuals are insured (and thus observed), the higher the likelihood of having a coded diagnosis (average number of days insured within each occupational group and time period are presented in Table 1). Predicted probabilities for prevalent T2D in each of the four time periods were then obtained using “Margins at means” for age and insurance duration. Thus in our analyses, predicted probabilities correspond to the adjusted prevalence rates and could be interpreted as the two-year prevalence rates of T2D given the age and insurance duration of the corresponding group and time period are at their mean values. Effect sizes were illustrated by prevalence ratios (PR) which would display more adequate effects compared to odds ratios when prevalence of the considered outcome is relatively high (> 10%) [43], which could be the case for some of the subgroups in this study. Using the post estimation command ‘nlcom’, we obtained PRs for T2D prevalence in the time period p4 compared to p1 (reference group p1) to examine trends based on the above described logistic regression analyses. This method from obtaining prevalence ratios from logistic regression has been suggested and advocated by several authors [44–46]. In order to deal with autocorrelation associated with the possibility of having individuals in several time periods, the models also corrected for within-cluster variation by using robust standard errors [47].

**Table 1:** Population characteristics in the four time periods stratified by occupational sector

	**p1 (2012–2013)**							**p2 (2014–2015)**						
	**N(%)**	**T2D rate %**	**Female %**	**18–45 y %**	**Age M(sd) in 18–45 y**	**Age M(sd) in > 45 y**	**Days insured n(sd)**	**N(%)**	**T2D rate %**	**Female %**	**18–45 y %**	**Age M(sd) in 18–45 y**	**Age M(sd) in > 45 y**	**Days insured n(sd)**
**Agriculture**	35850 (4)	2,68	26	65	30 (8)	53 (6)	509 (284)	38662 (4)	2,81	26	64	30 (8)	53 (6)	509 (283)
**Extraction of raw material, production and manufacturing**	246768 (26)	4,36	18	60	31 (8)	53 (5)	670 (167)	250642 (25)	4,55	18	58	31 (8)	54 (6)	661 (178)
**Construction, architecture, measuring and building technology**	88314 (9)	4,41	3	56	32 (8)	53 (6)	673 (161)	90539 (9)	4,53	3	55	32 (8)	54 (6)	662 (178)
**Natural sciences, geography, information**	15658 (2)	3,66	21	70	31 (7)	53 (5)	690 (132)	16591 (2)	3,62	22	70	31 (7)	53 (6)	687 (136)
**Transport, logistics, protection and security**	202680 (21)	6,67	33	47	33 (8)	54 (6)	674 (162)	217831 (22)	6,76	33	47	33 (8)	54 (6)	658 (183)
**Commercial, trade, distribution and tourism**	116231 (12)	2,57	69	70	30 (8)	53 (5)	667 (165)	125799 (13)	2,71	69	68	30 (8)	53 (6)	662 (172)
**Corporate organization, accounting, law and administration**	97299 (10)	2,87	72	65	31 (8)	53 (6)	688 (134)	103698 (10)	2,93	72	64	31 (8)	53 (6)	680 (148)
**Health sector, social work, teaching & education**	130621 (13)	3,11	85	64	31 (8)	53 (5)	687 (136)	142312 (14)	3,26	85	63	31 (7)	54 (5)	681 (148)
**Humanities, culture and design**	13082 (1)	2,31	51	77	31 (7)	52 (6)	623 (219)	14287 (1)	2,34	51	77	31 (7)	52 (6)	624 (219)
**Overall**	946503	4,21	42	60	31 (8)	53 (6)	669 (168)	1000361	4,33	42	59	31 (8)	54 (6)	659 (180)

	**p3 (2016–2017)**							**p4 (2018–2019)**						
	**N(%)**	**T2D rate %**	**Female %**	**18–45 y %**	**Age M(sd) in 18–45 y**	**Age M(sd) in > 45 y**	**Days insured n(sd)**	**N(%)**	**T2D rate %**	**Female %**	**18–45 y %**	**Age M(sd) in 18–45 y**	**Age M(sd) in > 45 y**	**Days insured n(sd)**
**Agriculture**	41947 (4)	2,82	26	64	30 (8)	54 (6)	504 (286)	45221 (4)	2,94	26	63	30 (8)	54 (6)	511 (284)
**Extraction of raw material, production and manufacturing**	268108 (24)	4,37	18	59	31 (8)	54 (6)	648 (192)	293494 (23)	4,45	18	59	31 (8)	54 (6)	651 (189)
**Construction, architecture, measuring and building technology**	97544 (9)	4,32	3	56	31 (8)	54 (6)	645 (195)	108088 (9)	4,33	4	57	31 (8)	54 (6)	639 (201)
**Natural sciences, geography, information**	19574 (2)	3,32	23	71	31 (7)	53 (5)	674 (154)	24424 (2)	3,08	23	73	31 (7)	54 (5)	682 (139)
**Transport, logistics, protection and security**	241346 (21)	6,59	33	48	33 (7)	55 (6)	646 (197)	267921 (21)	6,69	32	50	33 (7)	55 (6)	646 (194)
**Commercial, trade, distribution and tourism**	142081 (13)	2,78	69	66	30 (7)	53 (5)	655 (180)	158912 (12)	3,05	67	66	30 (7)	54 (5)	665 (166)
**Corporate organization, accounting, law and administration**	126591 (11)	2,82	73	62	31 (7)	54 (6)	658 (170)	154404 (12)	2,88	73	62	31 (7)	54 (6)	676 (145)
**Health sector, social work, teaching & education**	172035 (15)	3,23	84	63	31(7)	54 (5)	667 (163)	207845 (16)	3,28	84	63	31 (7)	54 (6)	678 (146)
**Humanities, culture and design**	16640 (2)	2,42	53	75	31(7)	53 (6)	623 (219)	19264 (2)	2,57	55	74	31 (7)	53 (6)	644 (196)
**Overall**	1125866	4,19	43	59	31(8)	54(6)	647 (192)	1279573	4,25	44	59	31 (7)	54 (6)	654 (183)

All analyses were performed with the statistics software STATA version 16.0. Since the study deals with a whole population with large N rather than a sample, statistical significance based on p-values can be easily inferred and might be misleading [48]. Therefore, we used confidence intervals to infer statistical significance instead.

## Results

The study population included 946.503, 1.000.361, 1.125.866 and 1.279.573 employed individuals in p1, p2, p3 and p4, respectively. Average age ranged between 30–33 years in the younger age group, and 52–55 in the older age group. While the majority were men in the occupational sectors: “Agriculture”, “Extraction of raw material, production and manufacturing”, “Construction, Architecture, measuring and building technology”, “Natural Sciences, geography and information” and “Transport, logistics protection and security”, the majority were females in the other occupational sectors. For example, 85% were women in the occupational sector “Health sector, social work, teaching and education”. Moreover, a shift from production based occupations towards more service and science based occupations appears to be taking place. For example, there is a reduction in the proportion of individuals working in the sector “Extraction of raw material, production and manufacturing” between p1 (26%) and p4 (23%). At the same time, the proportions of individuals working in sectors: “Natural Sciences, geography and information”, “Corporate organization, accounting, law and administration” and “Health sector, social work, teaching & education” have increased over the observed periods. Detailed study population demographic characteristics are displayed in Table 1. Additional socioeconomic characteristics that were not part of the analyses in this study are found in Additional File 1.

### T2D prevalence among different occupational sectors and age groups

**Fig. 1:**
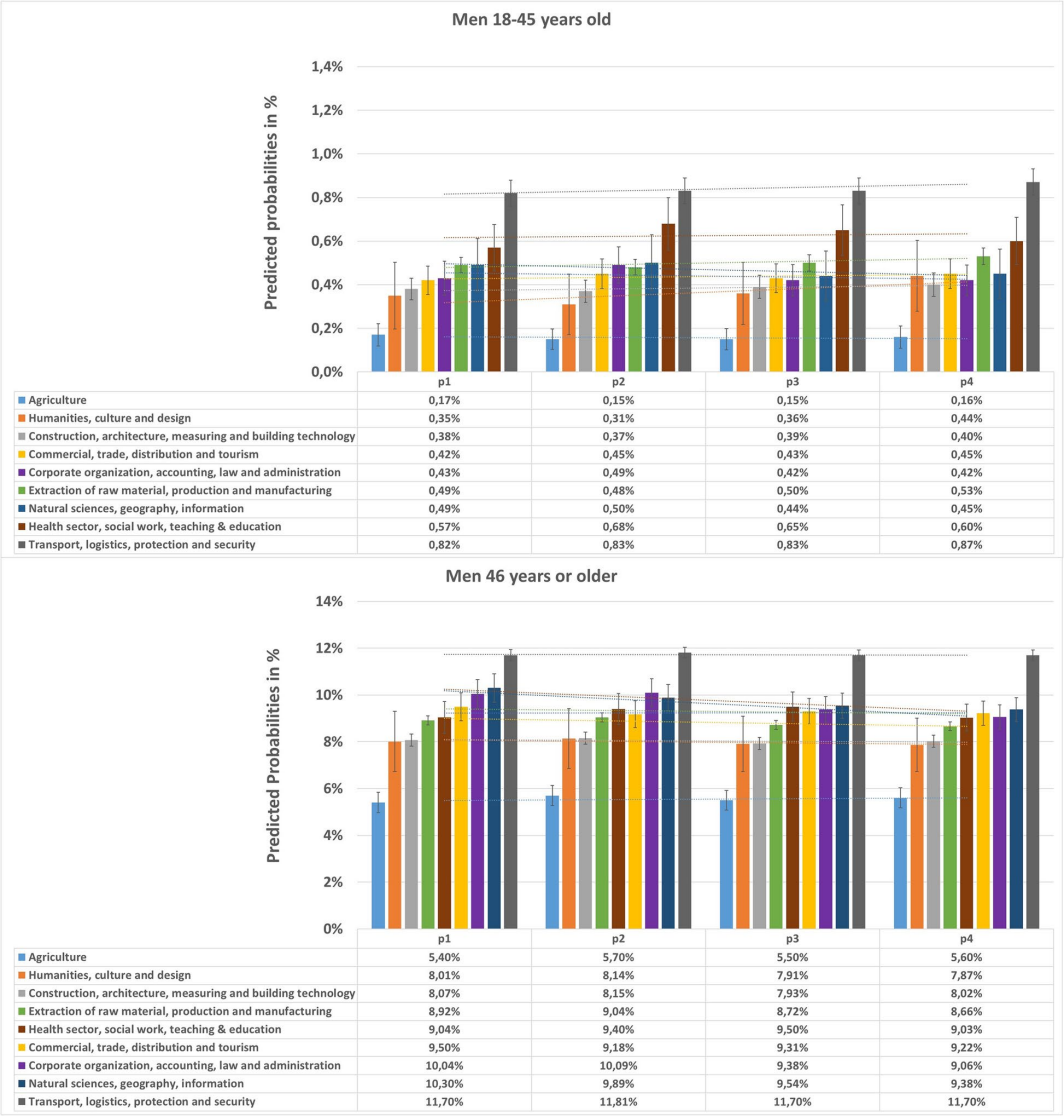
Predicted probabilities and 95% confidence intervals for prevalent T2D in different occupational sectors in *men*, stratified by two age groups. Estimated by logistic regression analyses adjusting for age within each age group and insurance duration. Corrected for within cluster variation using standard robust errors. p1(2012–2013), p2(2014–2015), p3(2016–2017), p4(2018–2019)

**Fig. 2:**
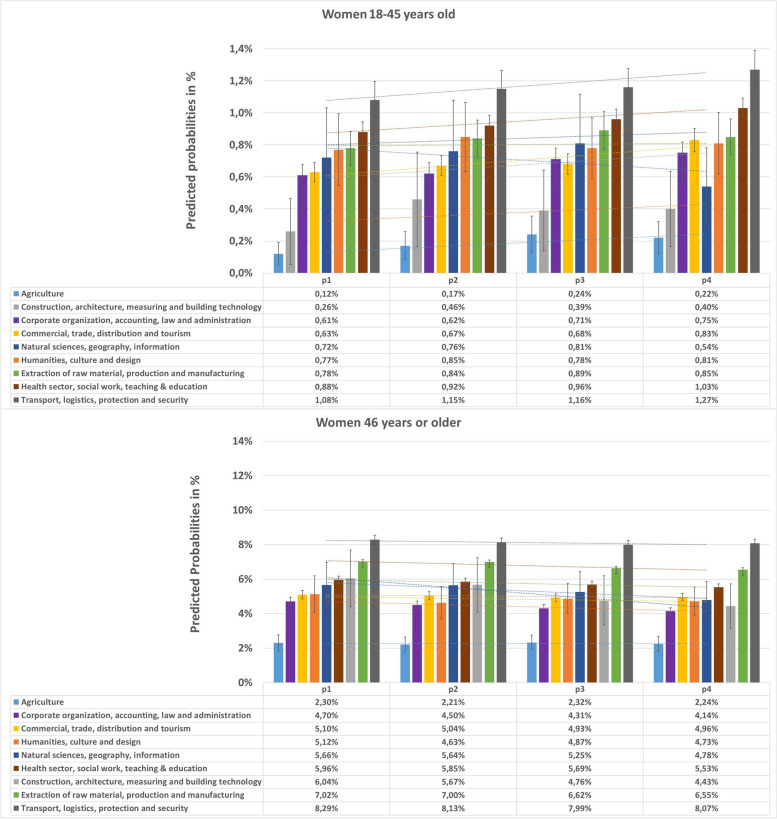
Predicted probabilities and 95% confidence intervals for prevalent T2D in different occupational sectors in *women*, stratified by two age groups. Estimated by logistic regression analyses adjusting for age within each age group and insurance duration. Corrected for within cluster variation using standard robust errors. p1(2012–2013), p2(2014–2015), p3(2016–2017), p4(2018–2019)

Employed men (Fig. 1) and women (Fig. 2) working in the occupational sector “Agriculture” had by far the lowest predicted probabilities for T2D prevalence. This applied to both examined age groups, despite a low T2D prevalence in the younger age group in absolute terms. On the other hand, the occupational sector “Transport, logistics, protection and security” had the highest predicted probabilities for T2D prevalence, which also applied to both genders and age groups. Among women and men of the younger age group, the occupational sector “Health sector, social work, teaching and education” ranked second for the highest prevalence of T2D among occupational sectors. Among the other occupational sectors that appeared to rank in the middle, different rankings were observed by gender, but differences among the sectors were not very pronounced.

### Trends of T2D prevalence among different occupational sectors and age groups

**Fig. 3:**
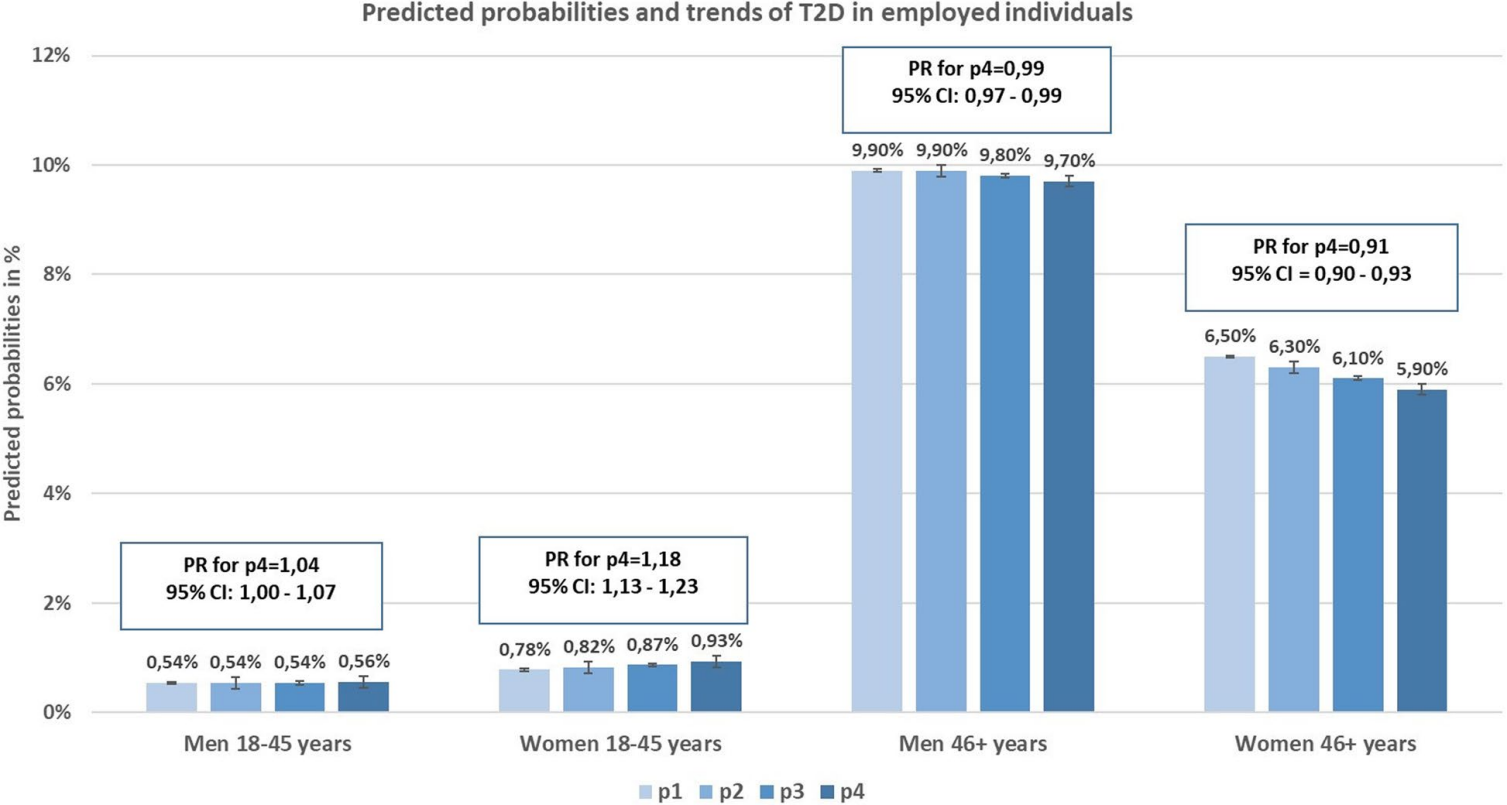
Bars: Predicted probabilities and 95% confidence intervals for T2D in the four time periods. Boxes: PRs (prevalence ratios) for prevalent T2D in p4 (2018–2019) compared to p1 (2012–2013). Based on a logistic regression analysis with T2D prevalence as the dependent variable and time period as the main independent variable. Adjusted for age within each age group and insurance duration. Corrected for within cluster variation using standard robust errors. p1(2012–2013), p2(2014–2015), p3(2016–2017), p4(2018–2019)

**Fig. 4:**
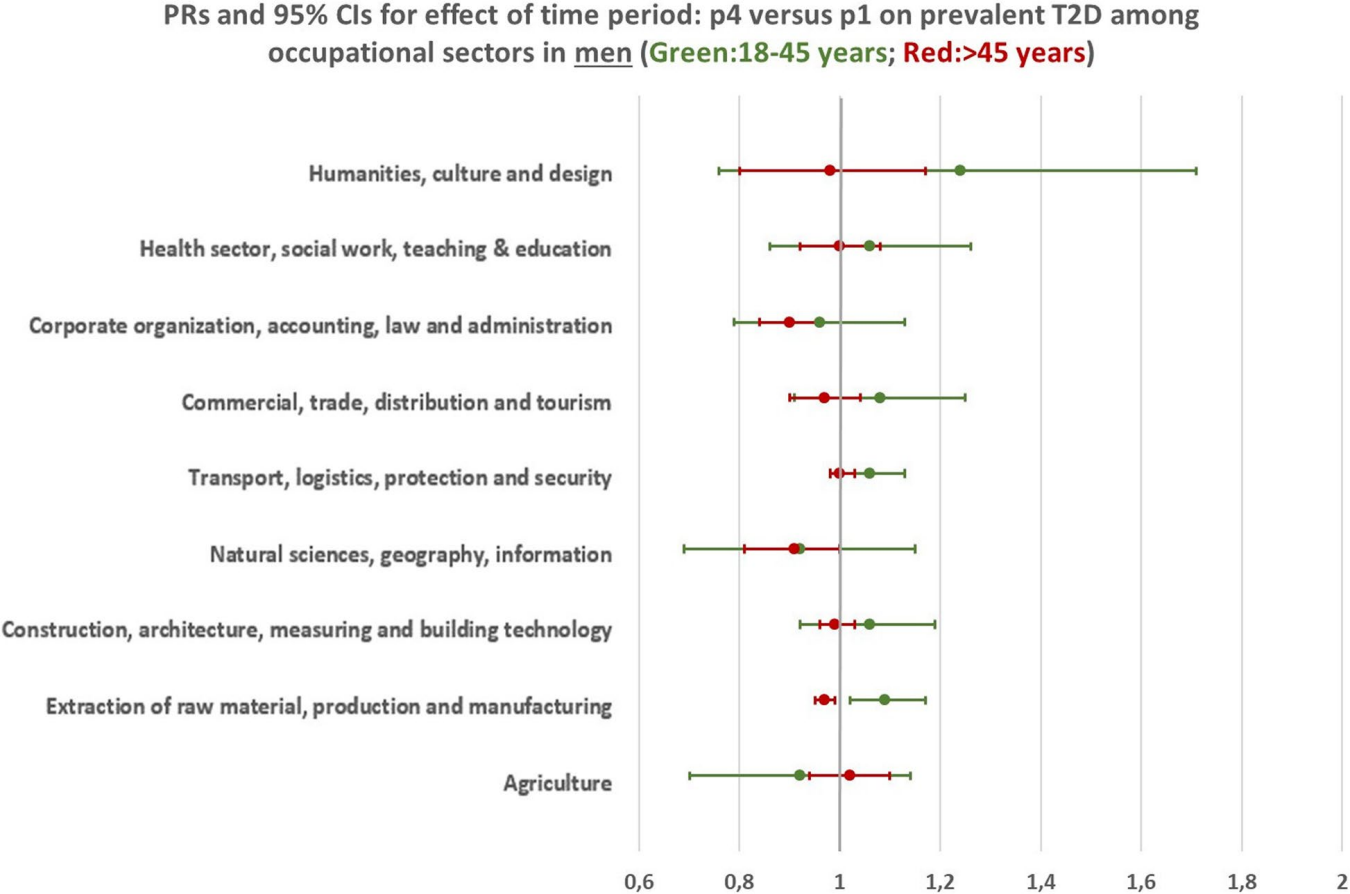
PRs (prevalence ratios) and confidence intervals for having prevalent T2D in p4 (2018–2019) compared to p1 (2012–2013) in men, based on a logistic regression analysis with T2D prevalence as the dependent variable and time period as the main independent variable. Adjusted for age within each age group and insurance duration. Corrected for within cluster variation using standard robust errors. p1(2012–2013), p4(2018–2019)

**Fig. 5:**
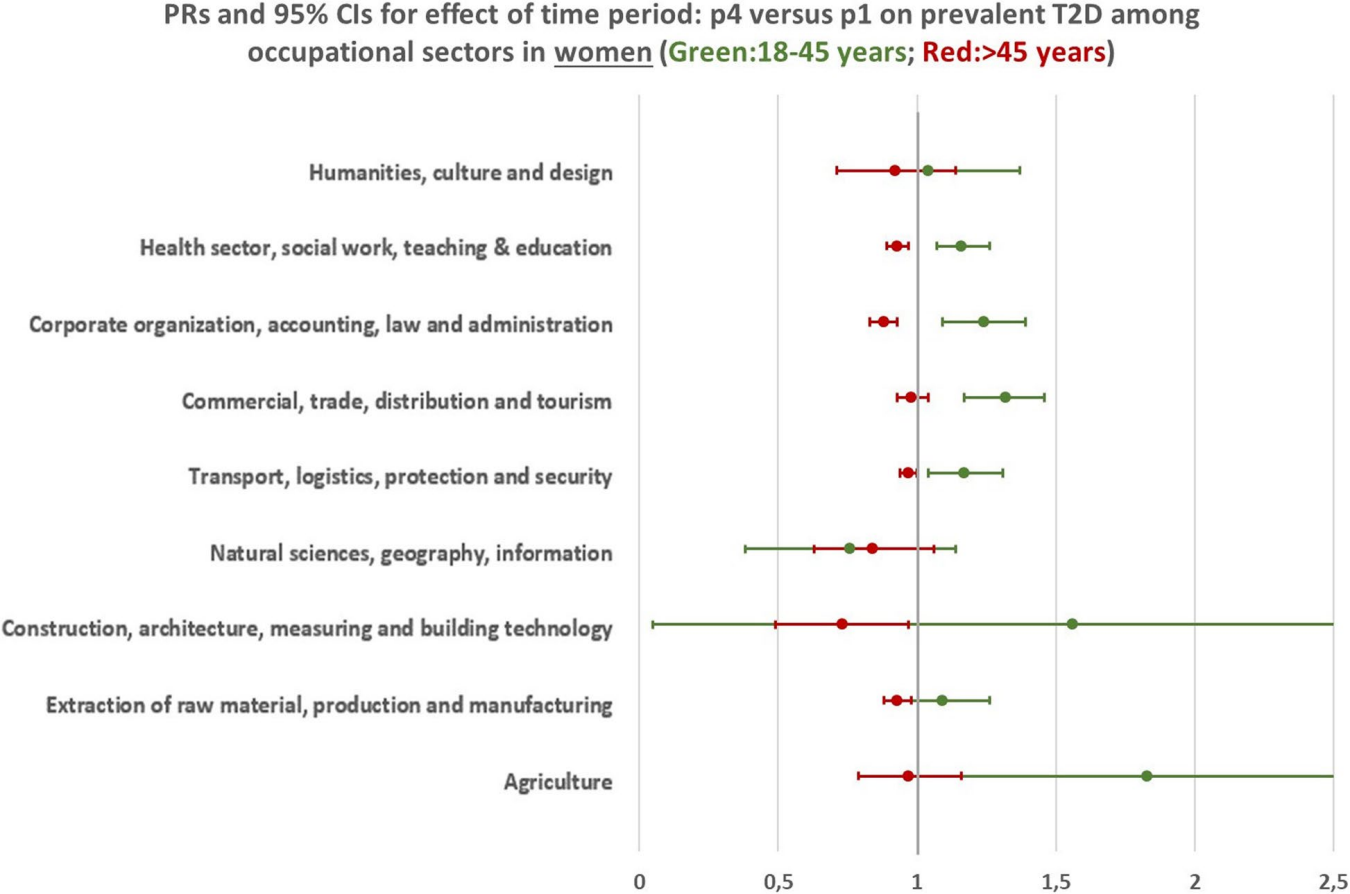
PRs (prevalence ratios) and confidence intervals for having prevalent T2D in p4 (2018–2019) compared to p1 (2012–2013) in women, based on a logistic regression analysis with T2D prevalence as the dependent variable and time period as the main independent variable. Adjusted for age within each age group and insurance duration. Corrected for within cluster variation using standard robust errors. p1(2012–2013), p4(2018–2019)

When considering the whole employed population, differential trends were observed among the two examined age groups. Results indicate a tendency towards a rising trend of T2D prevalence in the age group 18–45 years which was statistically significant in women (Men: PR = 1,04, 95% CI (1,00 – 1,07); Women: PR = 1,18, 95%CI (1,13 – 1,23)), and significantly declining trend for the age group 46 + years (Men: PR = 0,99, 95% CI (0,97 – 0,99); Women: PR = 0,91, 95%CI (0,90 – 0,93)) (Fig. 3). When stratifying the analyses by occupational sectors, significant trends could be observed only for some specific sectors. In men, statistically significant trends were found for the occupational sector “Extraction of raw material, production and manufacturing” with a T2D prevalence that was 9% greater in p4 compared to p1 (PR = 1,09, 95%CI (1,02 – 1,17)) for the younger age group, and 3% lower in the older group (PR = 0,97, 95%CI (0,95 – 0,99)) (Fig. 4 & Additional File 2). In women of the younger age group, a significant rise in the trend of T2D prevalence was shown for the occupational sectors “Health sector, social work, teaching and education”(PR = 1,16, 95%CI (1,07 – 1,26)), “Corporate organization, accounting, law and administration” (PR = 1,24, 95%CI (1,09 – 1,39)), “Commercial, trade, distribution and tourism” (PR = 1,32, 95%CI (1,17 – 1,46)) and “Transport, logistics, protection and security” (PR = 1,17, 95%CI (1,04 – 1,31)). On the other hand, women in the older age group working in the sectors “Health sector, social work, teaching and education” (PR = 0,93, 95%CI (0,89 – 0,97)), “Corporate organization, accounting, law and administration” (PR = 0,88, 95%CI (0,83 – 0,97)), “Extraction of raw material, production and manufacturing” (PR = 0,93, 95%CI (0,88 – 0,98)) and “Construction, architecture, measuring and building technology” (PR = 0,73, 95%CI (0,49 – 0,97)) had a significantly lower T2D prevalence in p4 compared to p1 (Fig. 5 & Additional File 2). All other temporal changes were not statistically significant. Thus, the results indicate the tendency for a rising trend in T2D prevalence in the younger age group and a declining trend in the older age group.

## Discussion

This study investigated occupational sector differences in T2D prevalence for the employed population and examined trends thereof between 2012 and 2019.

### Differences among occupational sectors

The results showed clear differences among occupational sectors. Individuals working in the sector of agriculture have by far the lowest predicted probabilities for T2D, while those working in the sector “Transport, logistics, protection and security” have the highest predicted probabilities, being 2–4 times higher compared to “Agriculture”. These differences are not age standardized among the different occupational sectors, as we aimed to provide information about the vulnerability level of the occupational sectors regardless of age and other specific characteristics. However, the mean age was quite similar among occupational sectors that belong to the same gender and age group. The few existing studies in the literature partly share similar results. A Swedish study performed with data from national registers including all Swedish citizens indicated that professional drivers had a three times higher risk for T2D compared to other occupational groups that belong to the health and education sectors [19]. Similarly, an Australian study showed that the occupation group of machinery operators and drivers had the highest prevalence and risk for T2D among other occupational groups [21]. Moreover, an American analysis based on 90,000 interviews with working individuals also concluded that diabetes rates were highest among transportation workers [20]. Our study also added that this result applies even when stratifying the working population by gender and two age groups.

However, dissimilar to our study, the above-mentioned studies showed that individuals working in the health sector had the lowest T2D rates. Our gender and age group stratified analyses showed however that men and women of the younger age group working in the occupational sector of “Health sector, social work, teaching and education” had the second highest predicted probabilities for T2D. One explanation could lie behind the amount of stress and workload associated with occupations of this sector. A recent German review presented evidence on the association between the relatively high working time and sleep disorders in individuals working in health care and effects on mental and physical health outcomes such as cardiovascular diseases and depression [49], which are in turn established risk factors for T2D [50–52]. Similarly, stress among teachers in the education sector as depicted by relatively high levels of mental illness and psychosomatic outcomes is pronounced in Germany [53], which could also explain the higher predicted probabilities for T2D in this group.

The results also showed that individuals of both genders and age groups working in the occupational sector of “Agriculture” have by far the lowest predicted probabilities for T2D. This finding indicates that occupational inequalities in T2D do not necessarily entail the socioeconomic level as classically depicted, as most jobs that belong to this sector are not required to have finished a specific education. Moreover, it is reported that in Europe, 68% of individuals working in the agriculture sector belong to the low skilled category, which is by far the highest proportion compared to other occupational sectors [13]. In fact, tasks in most jobs belonging to this sector require physical movement and a certain level of fitness leading to health selection, which might explain the lower predicted probabilities for T2D. Nevertheless, it remains an open question whether it is the protective effect of physical activity that leads to lower predicted probabilities for T2D in this sector, or rather a selection effect due to the inability to work in this field when disability associated with chronic diseases is present.

### Trends of T2D among occupational sectors in different age groups

The analyses showed differential trends of T2D prevalence for the two considered age groups of the employed population. While there was a significant increase in the prevalence of T2D in the age group 18–45 years between 2012–13 and 2018–19, T2D prevalence decreased for the age group 46 + years. Even though marginal (most probably due to the relatively short observation period), the temporal development was significant in both age groups. The increase in the predicted probabilities for T2D in the younger age group is in line with the abundant evidence on the overall increasing risk for T2D [3, 4]. The results of this study have added that the rising trend also applies to the working population of younger age, a finding essential to inform public health policy for promoting occupational health. Nevertheless, the finding on the declining trend of T2D in the older age group should be regarded with attention. Several studies from Germany and other European countries have indicated a rising trend in disability and functional limitations among middle aged or older adults [54–56]. Moreover, a German study found that prevalence rates of functional limitations and disability were higher among unemployed individuals in employment age compared to employed ones [54]. Furthermore, previous research using the same claims data of this study showed that probabilities for most T2D comorbidities are pronouncedly increasing in middle age individuals [5] as well as specifically in employed individuals with T2D, reflecting an expansion of morbidity in this population [41]. This could imply that older working individuals might suffer from more disability associated with comorbidities and would thus exit the labor market. This “healthy worker bias” could explain the declining T2D trend in the age group of employed individuals analyzed in this study. In fact, the generally rising trends of T2D prevalence in the younger age group is alarming and highlights potential hazards of lifestyle risk factors associated with digitalization and the change in work contexts.

When stratifying the analyses by occupational sector, differential and gender specific trends could be observed, and only some occupational sectors displayed statistically significant temporal differences over time. The subgroup of younger men working in the sector “Extraction of raw material, production and manufacturing” exhibited a statistically significant rise in the trend of T2D, while this applied to women in the occupational sectors “Health sector, social work, teaching & education”, “Corporate organization, accounting, law and administration”, “Commercial, trade, distribution and tourism” and “Transport, logistics, protection and security”. This highlights the importance of subgroup stratification when the data permits in order to capture specific vulnerable groups at which prevention intervention should be targeted. Nevertheless, longitudinal evidence on T2D prevalence is lacking, specifically in working populations or among occupational groups. Thus, no supporting evidence for the trend results of this study exists, and there is a need to further investigate trends of chronic diseases among different occupations, as this is a key factor for improving occupational health.

Nevertheless, it is important to note that given the analyses of the study, causality between occupational sectors and T2D prevalence cannot be inferred, as potential confounders or mediators were not considered in the analyses. In fact, this was also not the aim of the study, as the study aimed to display different vulnerability levels among different occupational sectors, and further analyses that demonstrate a deeper investigation of occupations within occupational sectors are needed to examine potential mediating factors. This is especially relevant because different occupations are associated with different exposures that can shape the metabolic risk profiles of working individuals. For example, while working in a transport occupation can be associated with sedentary behavior and a prolonged siting time [57], those working in the health sector might be more prone to risk factors associated with shift work, sleeping disorders and stress [26–28, 31]. Besides that, individuals working in construction or extraction of raw materials occupations could be more likely to be exposed to chemicals, which is also among the occupational exposures that shape a metabolic risk profile [29]. Moreover, while occupational sectors examined in the study encompass occupations with variant socioeconomic levels, the role of SES in the observed differences and trends cannot be fully ruled out. Therefore, future studies should also consider examining socioeconomic status as a potential mediating factor.

Lastly, as was observed in the results illustrating the characteristics of the study population over the four time periods, there appears to be a shift from production and manual based occupations towards service and science based occupations has been taking place over time. This illustrates the change in the work structure that has been occurring in the last decades in Europe, partly due to the so labelled “Megatrends” such as digitalisation and use of information and communication technologies [13]. This could also play a role in the previously reported trends of prolonged sitting time in the working age population [11] and the temporal increase in insufficient physical activity especially in western countries [58], as well as the rising obesity trends in Germany [12]. Thus, the changing work-environments amplify warning signs for the increase in T2D prevalence and highlight the need for targeting occupational contexts when planning prevention interventions.

### Implications

Our results point towards occupational sector differences in the prevalence and the trends of T2D, which implies that employed individuals could benefit from occupation-tailored interventions for T2D prevention and management. Specifically, the occupational sectors “Transport, logistics, protection and security” and “Health sector, social work, teaching & education” should be focused upon by investigating specific occupations within the sectors as they appeared to be the most affected compared to other sectors. Moreover, examining the level of severity of T2D among different occupation groups would also provide evidence on vulnerability, as the extent to which T2D complications and disability would be developed could differ among individuals working in different sectors based on indicators like health literacy [59].

### Strengths and limitations

The study was based on claims data of a statutory health insured population in Lower Saxony, Germany. The data includes a large population and comprehensively all documented diagnoses, which limits selection bias associated with willingness and ability to participate in studies and loss to follow up. Moreover, this is the first German study investigating the prevalence and trends of T2D among different occupational sectors. Still, some limitations cannot be ruled out. First, given that the analyses were based on health insurance data, undiagnosed T2D were not taken into consideration. Evidence suggests that among other factors, younger age and being male are significantly associated factors with undiagnosed T2D [60]. Thus, T2D prevalence in the younger age groups and in men could have been underestimated in this study. Moreover, the socioeconomic structure of the AOKN differs somewhat from that of the general population in Germany [61], which might affect the generalizability of the results. In addition, due to data limitations associated with the change in the federal classification of occupations, the trends were observed over an eight-year period only, which allows for observing only marginal changes in T2D prevalence. Therefore, future studies should consider longer observational periods. In addition, health insurance data is associated with potential coding errors. Thus, the possibility of misclassifying T2D cannot be ruled out completely even after careful considerations in the classification. Moreover, it cannot be ruled out that changes in the frequency of diagnoses over time could have taken place as a result of changing guidelines. However, there is no evidence on diagnosis and coding changes that took place in the observed period. In addition, due to the several level stratification by age and gender, some subgroups were comparatively smaller resulting in relatively wider confidence intervals. Moreover, this study aimed to provide an overview on occupational sector differences as no evidence exists on that. Examining a narrower stratification of occupations was beyond the scope of the study, since considering the next level of the KldB2010 classification would have resulted in at least 37 occupational groups to examine and compare. Thus, future studies should focus on specific occupational sectors, especially the ones that appeared to be vulnerable, and examine specific occupations within the sectors that might differ in work contexts and associated risk factors. Future studies should also consider differentiating occupational position to depict the potential social inequalities within each occupational sector. Finally, we decided not to adjust for socioeconomic indicators such as income and education since the aim of the study was to capture vulnerable occupational sectors regardless of factors that could be associated. Considering SES indicators would be beyond the scope of the study and the discussion. Future studies should consider the vertical classification of socioeconomic factors, especially among occupational sectors that were shown to be more vulnerable.

## Conclusion

This study illustrated clear differences in the probability of having T2D among individuals working in different occupational sectors. It displayed that some occupational sectors like “Transport, logistics, protection and security” and “Health sector, social work, teaching & education” are more vulnerable than others and need to be focused upon in further research and when planning and implementing T2D prevention and management interventions. The rising trends in T2D prevalence among several occupational sectors points towards a possible increase in lifestyle risk factors that could also be associated with work contexts. Thus, a deeper investigation of occupational risk factors should be considered to identify starting points for T2D prevention.

### Supplementary Information

**Supplementary Material 1. **

**Supplementary Material 2. **

## Data Availability

The data underlying this study belong to the Allgemeine Ortskrankenkasse Niedersachsen (AOKN-General Local Health Insurance of Lower Saxony). The data are not publically available due to protection of data privacy of the insured individuals by the AOKN. Interested researchers can send data access requests to Dr. Jona Stahmeyer at the AOKN using the following e-mail address: Jona.Stahmeyer@aok.nds.de. The authors did not have any special access privilege.

## Abbreviations

AOKN: Allgemeine Ortskrankasse Niedersachsen
ICD-10-GM: German modified 10th version of the International Classification of Diseases
ISCO: International Standard Classification of Occupations
KldB2010: 2010 Classification of Occupations of the German Federal Employment Office
p1; p2; p3; p4: 2012-2013; 2014–2015; 2016–2017; 2018–2019
PR: Prevalence Ratio
T2D: Type 2 Diabetes
