# Supplementary material for: Type 2 diabetes in the employed population: do rates and trends differ among nine occupational sectors? An analysis using German health insurance claims data
Source: BMC Public Health. 2024 May 3;24:1231. doi: 10.1186/s12889-024-18705-5 (PMC11069294; doi:10.1186/s12889-024-18705-5)
Supplement: Supplementary file 1 — Supplementary Material 1. [file 12889_2024_18705_MOESM1_ESM.docx]

**Type 2 diabetes in the employed population: do rates and trends differ among nine occupational sectors? An analysis using German health insurance claims data**

Distributions of income* and school education** by time period and occupational sector

|  |  | **Agriculture** | **Extraction of raw material, production and manufacturing** | **Construction, architecture, measuring and building technology** | **Natural sciences, geography, information** | **Transport, logistics, protection and security** | **Commercial, trade, distribution and tourism** | **Corporate organization, accounting, law and administration** | **Health sector, social work, teaching & education** | **Humanities, culture and design** |
| --- | --- | --- | --- | --- | --- | --- | --- | --- | --- | --- |
| **Period 1 2012-2013** | **N** | **35850** | **246768** | **88314** | **15658** | **202680** | **116231** | **97299** | **130621** | **13082** |
|  | **Education** |  |  |  |  |  |  |  |  |  |
|  | Low | 27% | 34% | 44% | 18% | 38% | 26% | 9% | 15% | 10% |
|  | Middle | 19% | 34% | 28% | 34% | 24% | 45% | 42% | 44% | 29% |
|  | High | 6% | 7% | 5% | 32% | 5% | 14% | 31% | 23% | 36% |
|  | *missing* | 48% | 25% | 24% | 16% | 34% | 31% | 18% | 18% | 24% |
|  | **Income** |  |  |  |  |  |  |  |  |  |
|  | Lower | 21% | 17% | 11% | 14% | 31% | 43% | 29% | 36% | 23% |
|  | Middle | 11% | 15% | 11% | 9% | 18% | 13% | 13% | 16% | 13% |
|  | Higher | 16% | 47% | 52% | 59% | 27% | 13% | 38% | 26% | 27% |
|  | *missing* | 52% | 22% | 25% | 18% | 25% | 30% | 19% | 22% | 37% |
| **Period 2 2014-2015** | **N** | **38662** | **250642** | **90539** | **16591** | **217831** | **125799** | **103698** | **142312** | **14287** |
|  | **Education** |  |  |  |  |  |  |  |  |  |
|  | Low | 26% | 33% | 42% | 16% | 37% | 23% | 9% | 15% | 10% |
|  | Middle | 19% | 35% | 28% | 34% | 24% | 39% | 42% | 45% | 29% |
|  | High | 6% | 8% | 5% | 35% | 6% | 13% | 32% | 24% | 39% |
|  | *missing* | 48% | 24% | 25% | 15% | 33% | 26% | 17% | 16% | 23% |
|  | **Income** |  |  |  |  |  |  |  |  |  |
|  | Lower | 21% | 17% | 11% | 14% | 30% | 41% | 27% | 35% | 22% |
|  | Middle | 12% | 15% | 11% | 10% | 18% | 13% | 14% | 15% | 12% |
|  | Higher | 15% | 45% | 51% | 58% | 25% | 13% | 38% | 26% | 27% |
|  | *missing* | 52% | 23% | 26% | 18% | 28% | 32% | 21% | 23% | 38% |
| **Period 3 2016-2017** | **N** | **41947** | **268108** | **97544** | **19574** | **241346** | **142081** | **126591** | **172035** | **16640** |
|  | **Education** |  |  |  |  |  |  |  |  |  |
|  | Low | 25% | 31% | 40% | 14% | 36% | 22% | 7% | 14% | 8% |
|  | Middle | 19% | 35% | 28% | 34% | 25% | 38% | 42% | 45% | 28% |
|  | High | 7% | 9% | 6% | 38% | 7% | 14% | 35% | 26% | 42% |
|  | *missing* | 49% | 25% | 26% | 14% | 33% | 25% | 16% | 15% | 21% |
|  | **Income** |  |  |  |  |  |  |  |  |  |
|  | Lower | 21% | 16% | 11% | 12% | 29% | 40% | 25% | 33% | 22% |
|  | Middle | 13% | 15% | 12% | 10% | 18% | 14% | 13% | 15% | 12% |
|  | Higher | 14% | 44% | 49% | 57% | 23% | 14% | 37% | 26% | 29% |
|  | *missing* | 52% | 25% | 28% | 21% | 29% | 33% | 25% | 26% | 37% |
| **Period 4 2018-2019** | **N** | **45221** | **293494** | **108088** | **24424** | **267921** | **158912** | **154404** | **207845** | **19264** |
|  | **Education** |  |  |  |  |  |  |  |  |  |
|  | Low | 24% | 30% | 37% | 13% | 36% | 22% | 7% | 13% | 8% |
|  | Middle | 20% | 34% | 27% | 33% | 24% | 37% | 42% | 45% | 27% |
|  | High | 7% | 9% | 7% | 41% | 7% | 14% | 36% | 27% | 45% |
|  | *missing* | 49% | 26% | 30% | 13% | 34% | 26% | 15% | 15% | 20% |
|  | **Income** |  |  |  |  |  |  |  |  |  |
|  | Lower | 21% | 16% | 12% | 12% | 28% | 40% | 25% | 33% | 22% |
|  | Middle | 14% | 15% | 13% | 9% | 19% | 14% | 14% | 15% | 13% |
|  | Higher | 15% | 45% | 48% | 59% | 24% | 15% | 40% | 28% | 32% |
|  | *missing* | 49% | 24% | 28% | 20% | 29% | 30% | 22% | 24% | 34% |

****Income*** refers to the individual income per person and is classified into three levels labeled as: Lower (<60% of the average German annual income (AGI)). Middle (60–80% of the AGI). Higher (>80 % of the AGI). More information on income in the AOKN data and related justifications can be found in (1).

*****Education*** refers to the highest achieved school-leaving certificate. It is classified into three levels as follows: Low (≤9 years of schooling, which corresponds to the German Hauptschulabschluss or no school diploma). Middle (10 years of schooling, which corresponds to the German Realschulabschluss). High (12–13 years of schooling, which corresponds to the German Abitur which is equivalent to a high school diploma).

1. Safieddine B, Sperlich S, Beller J, Lange K, Geyer S. Socioeconomic inequalities in type 2 diabetes comorbidities in different population subgroups: trend analyses using German health insurance data. Scientific Reports. 2023;13(1):10855.
