# Supplementary material for: Type 2 diabetes in the employed population: do rates and trends differ among nine occupational sectors? An analysis using German health insurance claims data
Source: BMC Public Health. 2024 May 3;24:1231. doi: 10.1186/s12889-024-18705-5 (PMC11069294; doi:10.1186/s12889-024-18705-5)
Supplement: Supplementary file 2 — Supplementary Material 2. [file 12889_2024_18705_MOESM2_ESM.docx]

**Type 2 diabetes in the employed population: do rates and trends differ among nine occupational sectors? An analysis using German health insurance claims data**

PRs (prevalence ratios) and 95% confidence intervals for the risk of having T2D in p4 (2018-2019) compared to p1 (2012-2013) in men and women of two age groups, based on a logistic regression analysis with T2D prevalence as the dependent variable and time period as the main independent variable. Adjusted for age within each age group and insurance duration. Corrected for within cluster variation using standard robust errors.

|  | **Men 18-45 years** | | | **Men > 45 years** | | | **Women 18-45 years** | | | **Women > 45 years** | | |
| --- | --- | --- | --- | --- | --- | --- | --- | --- | --- | --- | --- | --- |
|  | **N** | **PR** | **95% CI** | **N** | **PR** | **95% CI** | **N** | **PR** | **95% CI** | **N** | **PR** | **95% CI** |
| **Agriculture** | 75798 | 0,92 | 0,70-1,14 | 43303 | 1,02 | 0,94-1,10 | 27563 | 1,83 | 0,97-2,69 | 15016 | 0,97 | 0,79-1,16 |
| **Extraction of raw material, production and manufacturing** | 532627 | 1,09 | 1,02-1,17 | 341346 | 0,97 | 0,95-0,99 | 91148 | 1,09 | 0,92-1,26 | 93891 | 0,93 | 0,88-0,98 |
| **Construction, architecture, measuring and building technology** | 206960 | 1,06 | 0,92-1,19 | 165879 | 0,99 | 0,96-1,03 | 8123 | 1,56 | 0,05-3,06 | 3523 | 0,73 | 0,49-0,97 |
| **Natural sciences, geography, information** | 42362 | 0,93 | 0,69-1,15 | 16749 | 0,91 | 0,81-1,00 | 11866 | 0,76 | 0,38-1,14 | 5270 | 0,84 | 0,63-1,06 |
| **Transport, logistics, protection and security** | 327323 | 1,06 | 0,98-1,13 | 297062 | 1,00 | 0,98-1,03 | 118947 | 1,17 | 1,04-1,31 | 186446 | 0,97 | 0,94-1,00 |
| **Commercial, trade, distribution and tourism** | 131454 | 1,08 | 0,90-1,20 | 41601 | 0,97 | 0,90-1,04 | 233601 | 1,32 | 1,17-1,46 | 136367 | 0,98 | 0,93-1,04 |
| **Corporate organization, accounting, law and administration** | 90454 | 0,96 | 0,79-1,13 | 42058 | 0,90 | 0,84-0,96 | 213528 | 1,24 | 1,09-1,39 | 135952 | 0,88 | 0,83-0,97 |
| **Health sector, social work, teaching & education** | 69073 | 1,06 | 0,86-1,26 | 32430 | 1,00 | 0,92-1,08 | 343492 | 1,16 | 1,07-1,26 | 207818 | 0,93 | 0,89-0,97 |
| **Humanities, culture and design** | 22764 | 1,24 | 0,76-1,71 | 7306 | 0,98 | 0,80-1,17 | 25028 | 1,04 | 0,71-1,37 | 8175 | 0,92 | 0,71-1,14 |
